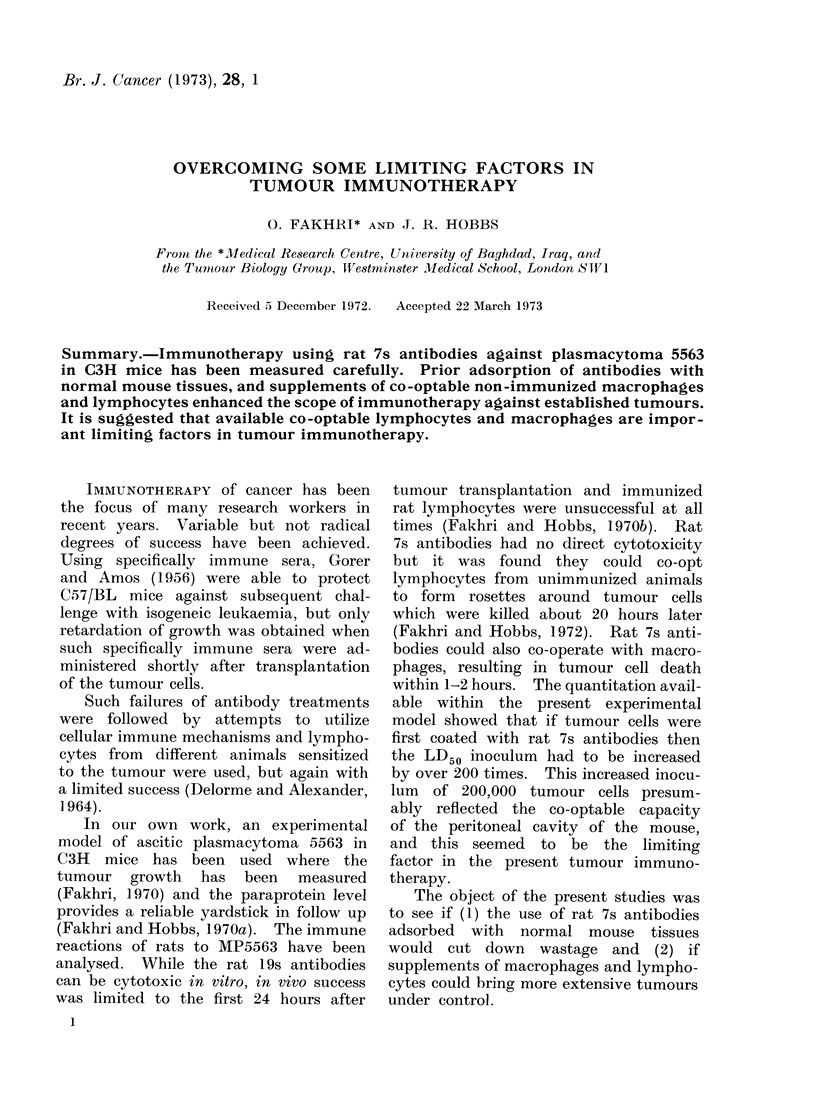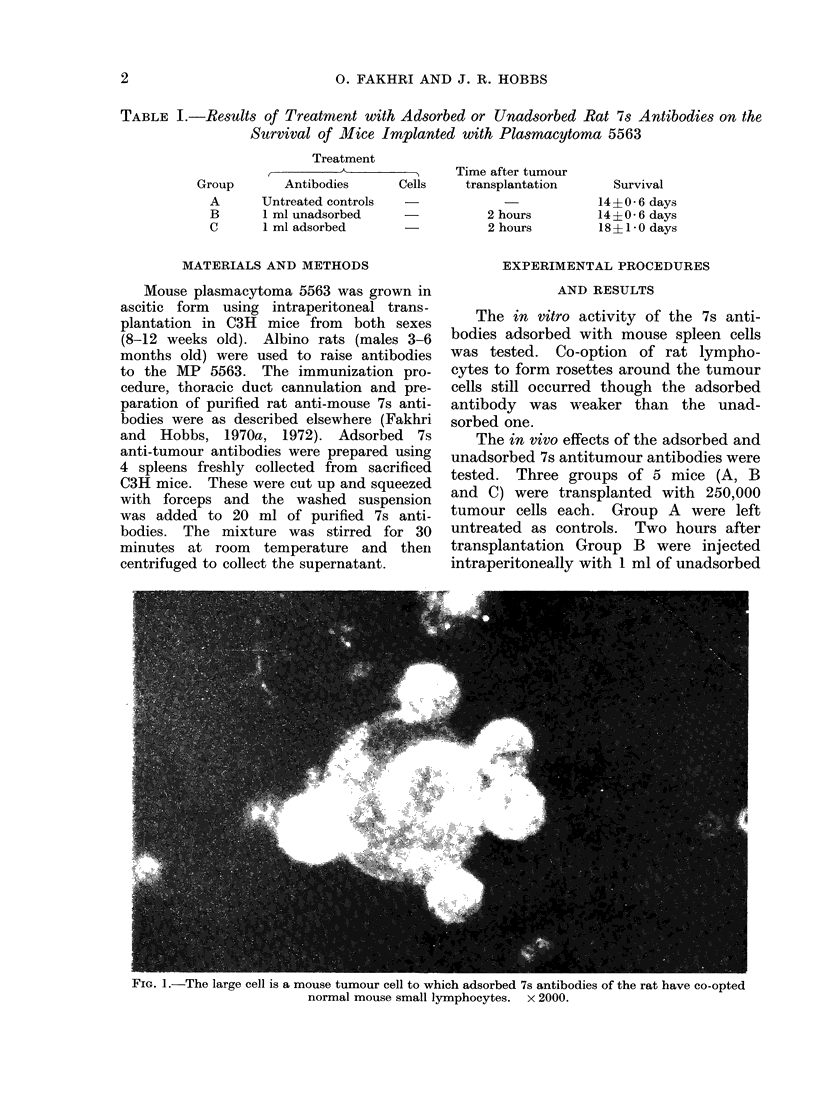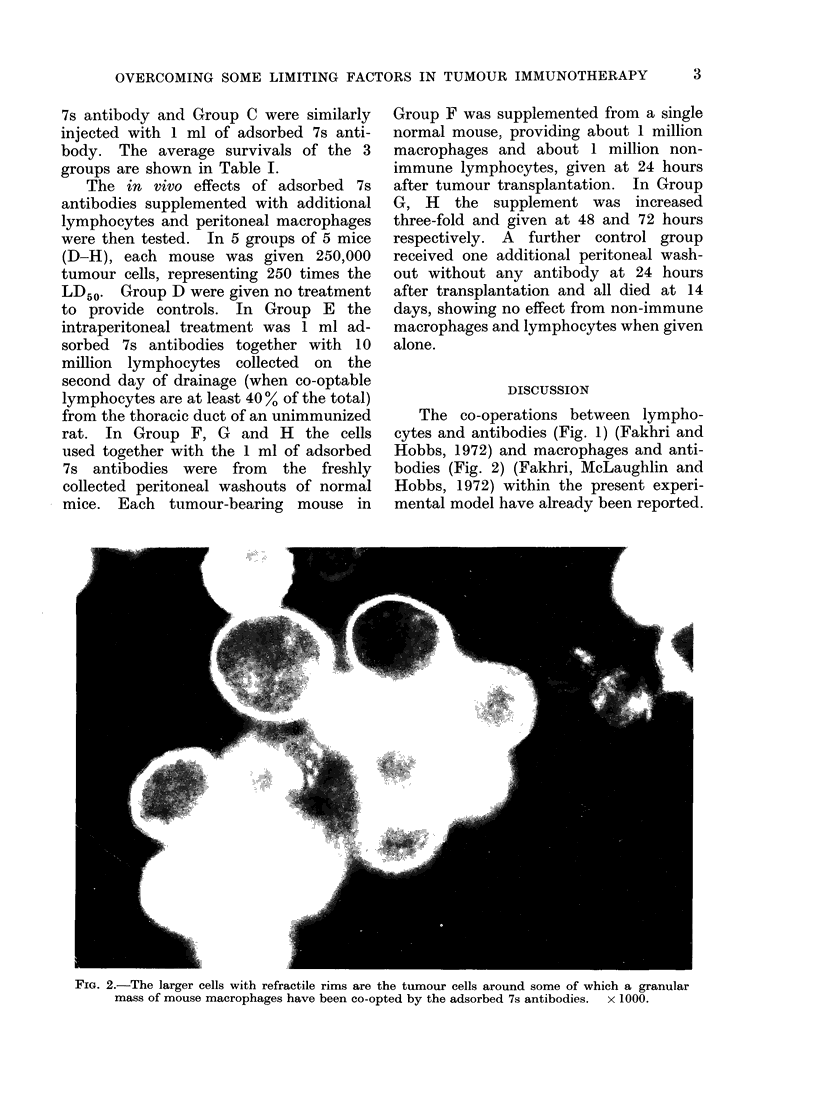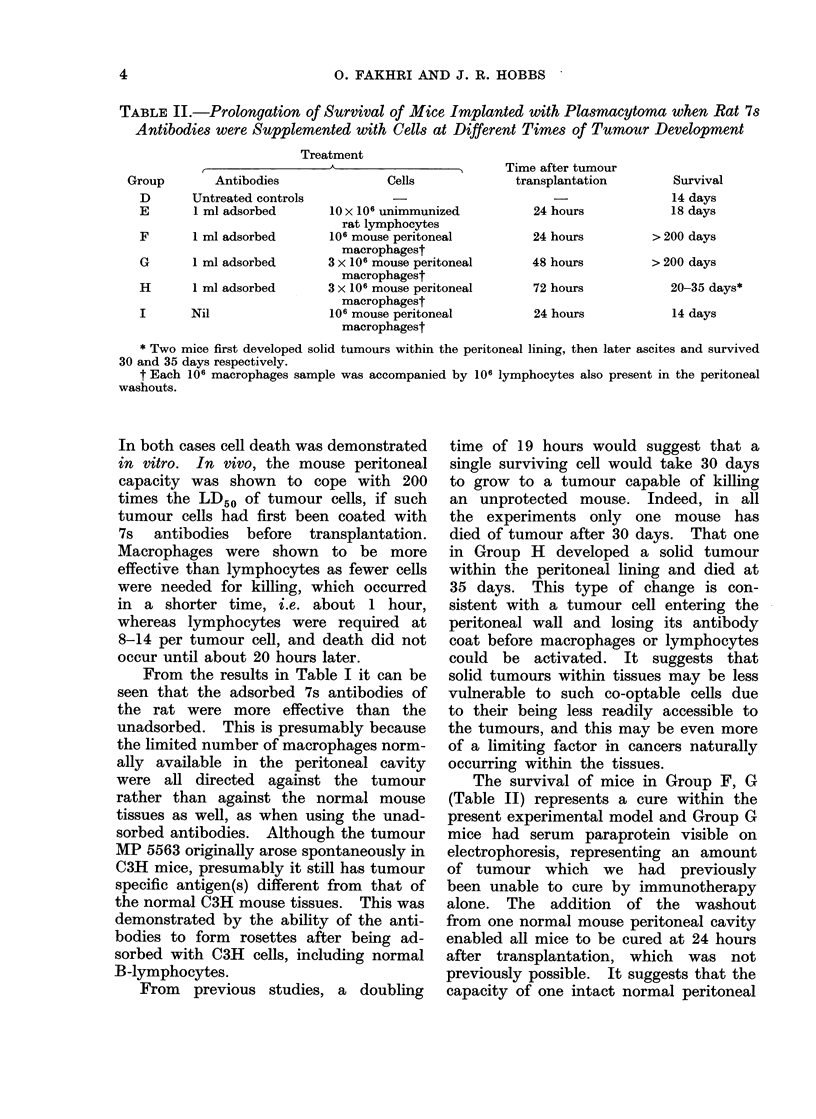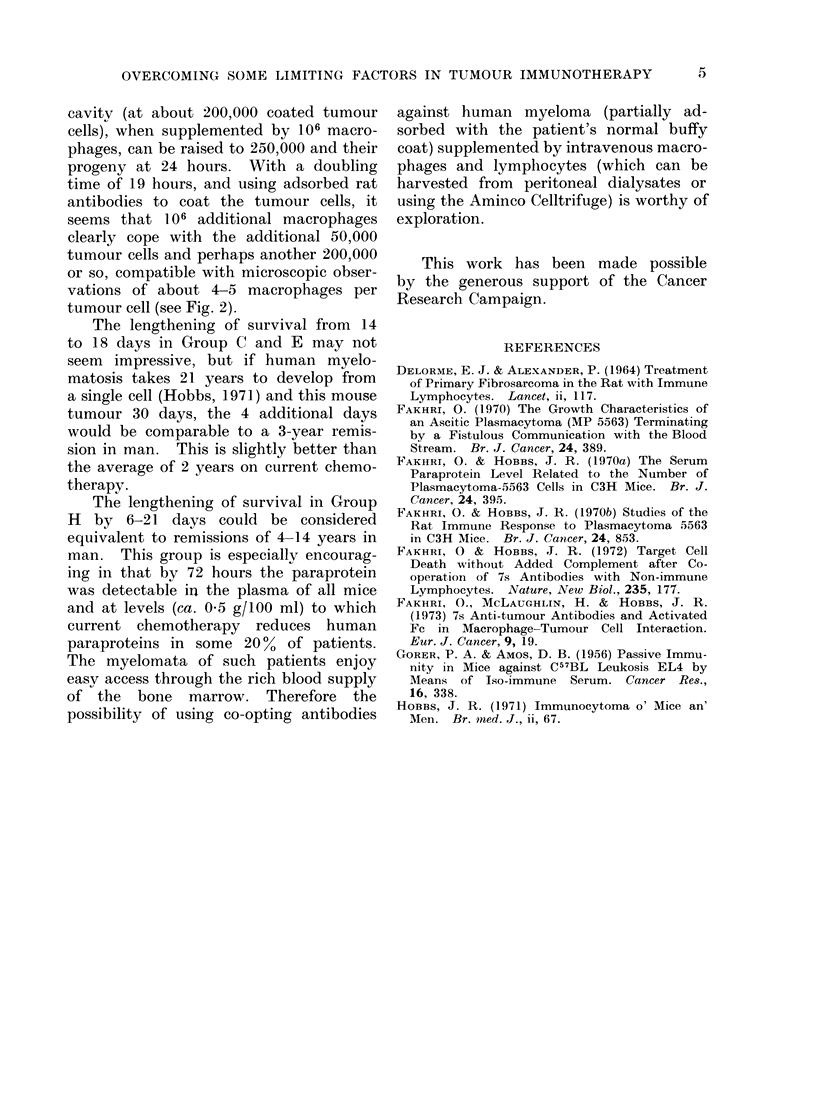# Overcoming some Limiting Factors in Tumour Immunotherapy

**DOI:** 10.1038/bjc.1973.64

**Published:** 1973-07

**Authors:** O. Fakhri, J. R. Hobbs

## Abstract

**Images:**


*Br. J. Cancer* (1973), **28**, 1

### OVERCOMING SOME LIMITING FACTORS IN TUMOUR IMMUNOTHERAPY

O. FAKHRI* AND J. R. HOBBS

*From the *Medical Research Centre, University of Baghdad, Iraq, and the Tumour Biology Group, Westminster Medical School, London SW1*

Received 5 December 1972. Accepted 22 March 1973

**Summary.—Immunotherapy using rat 7s antibodies against plasmacytoma 5563 in C3H mice has been measured carefully. Prior adsorption of antibodies with normal mouse tissues, and supplements of co-optable non-immunized macrophages and lymphocytes enhanced the scope of immunotherapy against established tumours. It is suggested that available co-optable lymphocytes and macrophages are important limiting factors in tumour immunotherapy.**

IMMUNOTHERAPY of cancer has been the focus of many research workers in recent years. Variable but not radical degrees of success have been achieved. Using specifically immune sera, Gorer and Amos (1956) were able to protect C57/BL mice against subsequent challenge with isogeneic leukaemia, but only retardation of growth was obtained when such specifically immune sera were administered shortly after transplantation of the tumour cells.

Such failures of antibody treatments were followed by attempts to utilize cellular immune mechanisms and lymphocytes from different animals sensitized to the tumour were used, but again with a limited success (Delorme and Alexander, 1964).

In our own work, an experimental model of ascitic plasmacytoma 5563 in C3H mice has been used where the tumour growth has been measured (Fakhri, 1970) and the paraprotein level provides a reliable yardstick in follow up (Fakhri and Hobbs, 1970*a*). The immune reactions of rats to MP5563 have been analysed. While the rat 19s antibodies can be cytotoxic *in vitro*, *in vivo* success was limited to the first 24 hours after tumour transplantation and immunized rat lymphocytes were unsuccessful at all times (Fakhri and Hobbs, 1970*b*). Rat 7s antibodies had no direct cytotoxicity but it was found they could co-opt lymphocytes from unimmunized animals to form rosettes around tumour cells which were killed about 20 hours later (Fakhri and Hobbs, 1972). Rat 7s antibodies could also co-operate with macrophages, resulting in tumour cell death within 1–2 hours. The quantitation available within the present experimental model showed that if tumour cells were first coated with rat 7s antibodies then the $LD_{50}$ inoculum had to be increased by over 200 times. This increased inoculum of 200,000 tumour cells presumably reflected the co-optable capacity of the peritoneal cavity of the mouse, and this seemed to be the limiting factor in the present tumour immunotherapy.

The object of the present studies was to see if (1) the use of rat 7s antibodies adsorbed with normal mouse tissues would cut down wastage and (2) if supplements of macrophages and lymphocytes could bring more extensive tumours under control.

TABLE I.—*Results of Treatment with Adsorbed or Unadsorbed Rat 7s Antibodies on the Survival of Mice Implanted with Plasmacytoma* 5563

| Group | Treatment: Antibodies | Treatment: Cells | Time after tumour transplantation | Survival |
|---|---|---|---|---|
| A | Untreated controls | — | — | $14 \pm 0 \cdot 6$ days |
| B | 1 ml unadsorbed | — | 2 hours | $14 \pm 0 \cdot 6$ days |
| C | 1 ml adsorbed | — | 2 hours | $18 \pm 1 \cdot 0$ days |

### MATERIALS AND METHODS

Mouse plasmacytoma 5563 was grown in ascitic form using intraperitoneal transplantation in C3H mice from both sexes (8–12 weeks old). Albino rats (males 3–6 months old) were used to raise antibodies to the MP 5563. The immunization procedure, thoracic duct cannulation and preparation of purified rat anti-mouse 7s antibodies were as described elsewhere (Fakhri and Hobbs, 1970*a*, 1972). Adsorbed 7s anti-tumour antibodies were prepared using 4 spleens freshly collected from sacrificed C3H mice. These were cut up and squeezed with forceps and the washed suspension was added to 20 ml of purified 7s antibodies. The mixture was stirred for 30 minutes at room temperature and then centrifuged to collect the supernatant.

### EXPERIMENTAL PROCEDURES AND RESULTS

The *in vitro* activity of the 7s antibodies adsorbed with mouse spleen cells was tested. Co-option of rat lymphocytes to form rosettes around the tumour cells still occurred though the adsorbed antibody was weaker than the unadsorbed one.

The *in vivo* effects of the adsorbed and unadsorbed 7s antitumour antibodies were tested. Three groups of 5 mice (A, B and C) were transplanted with 250,000 tumour cells each. Group A were left untreated as controls. Two hours after transplantation Group B were injected intraperitoneally with 1 ml of unadsorbed

FIG. 1.—The large cell is a mouse tumour cell to which adsorbed 7s antibodies of the rat have co-opted normal mouse small lymphocytes. ×2000.

7s antibody and Group C were similarly injected with 1 ml of adsorbed 7s antibody. The average survivals of the 3 groups are shown in Table I.

The *in vivo* effects of adsorbed 7s antibodies supplemented with additional lymphocytes and peritoneal macrophages were then tested. In 5 groups of 5 mice (D–H), each mouse was given 250,000 tumour cells, representing 250 times the $LD_{50}$. Group D were given no treatment to provide controls. In Group E the intraperitoneal treatment was 1 ml adsorbed 7s antibodies together with 10 million lymphocytes collected on the second day of drainage (when co-optable lymphocytes are at least 40% of the total) from the thoracic duct of an unimmunized rat. In Group F, G and H the cells used together with the 1 ml of adsorbed 7s antibodies were from the freshly collected peritoneal washouts of normal mice. Each tumour-bearing mouse in Group F was supplemented from a single normal mouse, providing about 1 million macrophages and about 1 million non-immune lymphocytes, given at 24 hours after tumour transplantation. In Group G, H the supplement was increased three-fold and given at 48 and 72 hours respectively. A further control group received one additional peritoneal washout without any antibody at 24 hours after transplantation and all died at 14 days, showing no effect from non-immune macrophages and lymphocytes when given alone.

### DISCUSSION

The co-operations between lymphocytes and antibodies (Fig. 1) (Fakhri and Hobbs, 1972) and macrophages and antibodies (Fig. 2) (Fakhri, McLaughlin and Hobbs, 1972) within the present experimental model have already been reported.

FIG. 2.—The larger cells with refractile rims are the tumour cells around some of which a granular mass of mouse macrophages have been co-opted by the adsorbed 7s antibodies. × 1000.

TABLE II.—*Prolongation of Survival of Mice Implanted with Plasmacytoma when Rat 7s Antibodies were Supplemented with Cells at Different Times of Tumour Development*

| Group | Treatment: Antibodies | Treatment: Cells | Time after tumour transplantation | Survival |
|---|---|---|---|---|
| D | Untreated controls | — | — | 14 days |
| E | 1 ml adsorbed | $10 \times 10^6$ unimmunized rat lymphocytes | 24 hours | 18 days |
| F | 1 ml adsorbed | $10^6$ mouse peritoneal macrophages† | 24 hours | > 200 days |
| G | 1 ml adsorbed | $3 \times 10^6$ mouse peritoneal macrophages† | 48 hours | > 200 days |
| H | 1 ml adsorbed | $3 \times 10^6$ mouse peritoneal macrophages† | 72 hours | 20–35 days* |
| I | Nil | $10^6$ mouse peritoneal macrophages† | 24 hours | 14 days |

* Two mice first developed solid tumours within the peritoneal lining, then later ascites and survived 30 and 35 days respectively.

† Each $10^6$ macrophages sample was accompanied by $10^6$ lymphocytes also present in the peritoneal washouts.

In both cases cell death was demonstrated *in vitro*. *In vivo*, the mouse peritoneal capacity was shown to cope with 200 times the $LD_{50}$ of tumour cells, if such tumour cells had first been coated with 7s antibodies before transplantation. Macrophages were shown to be more effective than lymphocytes as fewer cells were needed for killing, which occurred in a shorter time, *i.e.* about 1 hour, whereas lymphocytes were required at 8–14 per tumour cell, and death did not occur until about 20 hours later.

From the results in Table I it can be seen that the adsorbed 7s antibodies of the rat were more effective than the unadsorbed. This is presumably because the limited number of macrophages normally available in the peritoneal cavity were all directed against the tumour rather than against the normal mouse tissues as well, as when using the unadsorbed antibodies. Although the tumour MP 5563 originally arose spontaneously in C3H mice, presumably it still has tumour specific antigen(s) different from that of the normal C3H mouse tissues. This was demonstrated by the ability of the antibodies to form rosettes after being adsorbed with C3H cells, including normal B-lymphocytes.

From previous studies, a doubling time of 19 hours would suggest that a single surviving cell would take 30 days to grow to a tumour capable of killing an unprotected mouse. Indeed, in all the experiments only one mouse has died of tumour after 30 days. That one in Group H developed a solid tumour within the peritoneal lining and died at 35 days. This type of change is consistent with a tumour cell entering the peritoneal wall and losing its antibody coat before macrophages or lymphocytes could be activated. It suggests that solid tumours within tissues may be less vulnerable to such co-optable cells due to their being less readily accessible to the tumours, and this may be even more of a limiting factor in cancers naturally occurring within the tissues.

The survival of mice in Group F, G (Table II) represents a cure within the present experimental model and Group G mice had serum paraprotein visible on electrophoresis, representing an amount of tumour which we had previously been unable to cure by immunotherapy alone. The addition of the washout from one normal mouse peritoneal cavity enabled all mice to be cured at 24 hours after transplantation, which was not previously possible. It suggests that the capacity of one intact normal peritoneal cavity (at about 200,000 coated tumour cells), when supplemented by $10^6$ macrophages, can be raised to 250,000 and their progeny at 24 hours. With a doubling time of 19 hours, and using adsorbed rat antibodies to coat the tumour cells, it seems that $10^6$ additional macrophages clearly cope with the additional 50,000 tumour cells and perhaps another 200,000 or so, compatible with microscopic observations of about 4–5 macrophages per tumour cell (see Fig. 2).

The lengthening of survival from 14 to 18 days in Group C and E may not seem impressive, but if human myelomatosis takes 21 years to develop from a single cell (Hobbs, 1971) and this mouse tumour 30 days, the 4 additional days would be comparable to a 3-year remission in man. This is slightly better than the average of 2 years on current chemotherapy.

The lengthening of survival in Group H by 6–21 days could be considered equivalent to remissions of 4–14 years in man. This group is especially encouraging in that by 72 hours the paraprotein was detectable in the plasma of all mice and at levels (*ca.* 0·5 g/100 ml) to which current chemotherapy reduces human paraproteins in some 20% of patients. The myelomata of such patients enjoy easy access through the rich blood supply of the bone marrow. Therefore the possibility of using co-opting antibodies against human myeloma (partially adsorbed with the patient's normal buffy coat) supplemented by intravenous macrophages and lymphocytes (which can be harvested from peritoneal dialysates or using the Aminco Celltrifuge) is worthy of exploration.

This work has been made possible by the generous support of the Cancer Research Campaign.

### REFERENCES

DELORME, E. J. & ALEXANDER, P. (1964) Treatment of Primary Fibrosarcoma in the Rat with Immune Lymphocytes. *Lancet*, ii, 117.

FAKHRI, O. (1970) The Growth Characteristics of an Ascitic Plasmacytoma (MP 5563) Terminating by a Fistulous Communication with the Blood Stream. *Br. J. Cancer*, **24**, 389.

FAKHRI, O. & HOBBS, J. R. (1970*a*) The Serum Paraprotein Level Related to the Number of Plasmacytoma-5563 Cells in C3H Mice. *Br. J. Cancer*, **24**, 395.

FAKHRI, O. & HOBBS, J. R. (1970*b*) Studies of the Rat Immune Response to Plasmacytoma 5563 in C3H Mice. *Br. J. Cancer*, **24**, 853.

FAKHRI, O & HOBBS, J. R. (1972) Target Cell Death without Added Complement after Cooperation of 7s Antibodies with Non-immune Lymphocytes. *Nature, New Biol.*, **235**, 177.

FAKHRI, O., MCLAUGHLIN, H. & HOBBS, J. R. (1973) 7s Anti-tumour Antibodies and Activated Fc in Macrophage–Tumour Cell Interaction. *Eur. J. Cancer*, **9**, 19.

GORER, P. A. & AMOS, D. B. (1956) Passive Immunity in Mice against $C^{57}$BL Leukosis EL4 by Means of Iso-immune Serum. *Cancer Res.*, **16**, 338.

HOBBS, J. R. (1971) Immunocytoma o' Mice an' Men. *Br. med. J.*, ii, 67.